# CRISPR Technology and Its Emerging Applications

**DOI:** 10.1093/gpbjnl/qzaf034

**Published:** 2025-04-23

**Authors:** Xuejing Zhang, Dongyuan Ma, Feng Liu

**Affiliations:** Shandong Provincial Key Laboratory of Animal Cell and Developmental Biology, School of Life Sciences, Qilu Hospital (Qingdao), Cheeloo College of Medicine, Shandong University, Qingdao 266237, China; State Key Laboratory of Organ Regeneration and Reconstruction, Beijing Institute for Stem Cell and Regenerative Medicine, Institute of Zoology, University of Chinese Academy of Sciences, Chinese Academy of Sciences, Beijing 100101, China; Shandong Provincial Key Laboratory of Animal Cell and Developmental Biology, School of Life Sciences, Qilu Hospital (Qingdao), Cheeloo College of Medicine, Shandong University, Qingdao 266237, China; State Key Laboratory of Organ Regeneration and Reconstruction, Beijing Institute for Stem Cell and Regenerative Medicine, Institute of Zoology, University of Chinese Academy of Sciences, Chinese Academy of Sciences, Beijing 100101, China

**Keywords:** CRISPR, Genome editing, Lineage tracing, Gene function, Gene therapy

## Abstract

The discovery and iteration of clustered regularly interspaced short palindromic repeats (CRISPR) systems have revolutionized genome editing due to their remarkable efficiency and easy programmability, enabling precise manipulation of genomic elements. Owing to these unique advantages, CRISPR technology has the transformative potential to elucidate biological mechanisms and develop clinical treatments. This review provides a comprehensive overview of the development and applications of CRISPR technology. After describing the three primary CRISPR-Cas systems — CRISPR-associated protein 9 (Cas9) and Cas12a targeting DNA, and Cas13 targeting RNA — which serve as the cornerstone for technological advancements, we describe a series of novel CRISPR-Cas systems that offer new avenues for research, and then explore the applications of CRISPR technology in large-scale genetic screening, lineage tracing, genetic diagnosis, and gene therapy. As this technology evolves, it holds significant promise for studying gene functions and treating human diseases in the near future.

## Graphical abstract

**Figure:**
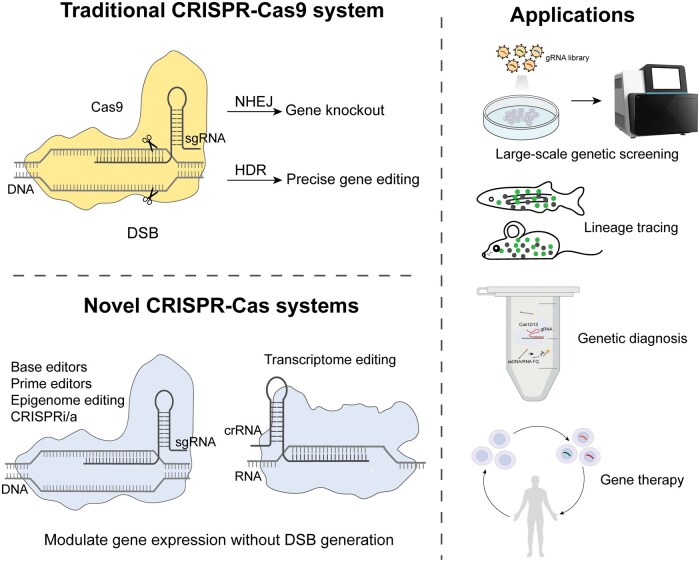

## Introduction

**Figure 1:**
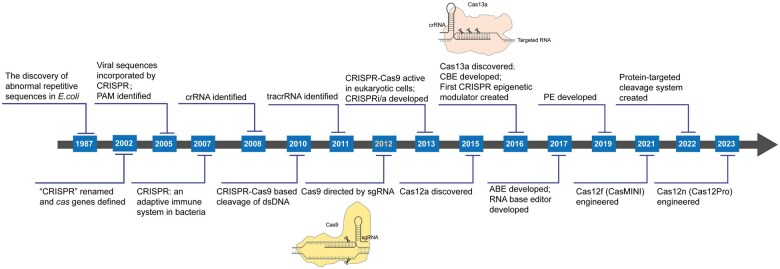
Development milestones of CRISPR-Cas technology In 1987, unusual DNA sequences were discovered in *E*. *coli* [9]. In 2002, repetitive sequences were renamed CRISPR, and *cas* genes were defined [10]. In 2005, CRISPR was found to incorporate viral sequences as part of its defense mechanism [11], and PAM was identified [12]. In 2007, CRISPR was recognized as the bacterial adaptive immune system [13]; In 2008, crRNA was identified [14]. In 2010, Cas9 was found to cleave dsDNA [15]. In 2011, tracrRNA was identified [16]. In 2012, Cas9 was shown to be directed by sgRNA to cleave dsDNA [3]. In 2013, CRISPR-Cas9 was found to be active in eukaryotic cells [4,5], and CRISPRi/a systems were developed [17]. In 2015, Cas12a was discovered [18]. In 2016, Cas13a was discovered to target RNA [19], CBE was developed [20], and the first CRISPR epigenetic modulator was created [21]. In 2017, ABE and RNA base editors were developed [22,23]. In 2019, PE was developed [24]. In 2021, Cas12f (CasMINI) was engineered as a miniature CRISPR protein [25]. In 2022, protein-targeted cleavage system was created [26,27]. In 2023, Cas12n (Cas12Pro) was engineered as another miniature CRISPR protein [28]. *E*. *coli*, *Escherichia coli*; CRISPR, clustered regularly interspaced short palindromic repeats; Cas, CRISPR-associated protein; PAM, protospacer adjacent motif; crRNA, CRISPR RNA; dsDNA, double-stranded DNA; tracrRNA, *trans*-activating crRNA; sgRNA, single-guide RNA; CRISPRi/a, CRISPR interference/activation; CBE, cytosine base editor; ABE, adenine base editor; PE, prime editor.

Genome editing is a technology that can accurately recognize the specific sequence within the genome, and then modify the targeted sequence to achieve different objectives [1]. Over the past two decades, several types of genome editing technologies, such as zinc finger nucleases (ZFNs), transcription activator-like effector nucleases (TALENs), and the relatively recent clustered regularly interspaced short palindromic repeats (CRISPR) systems, were successively introduced. Owing to the complex design and generation of ZFNs and TALENs, their applications in genome editing are limited [2]. In 2012, Emmanuelle Charpentier and Jennifer Doudna’s team collaborated to demonstrate that CRISPR-associated protein 9 (Cas9) nuclease can be guided by single-guide RNA (sgRNA), which is constituted of CRISPR RNA (crRNA) and *trans*-activating crRNA (tracrRNA), to induce a double-strand break (DSB) on the target sequence [3]. Afterward, several studies published in early 2013 found that the modified CRISPR-Cas9 system was adapted for genome editing in eukaryotic organisms [4–7]. Compared to ZFNs and TALENs, the programmability, specificity, and versatility of CRISPR-Cas systems have led to a revolutionary shift in genome editing technologies [8]. Since the CRISPR-Cas technology was developed, it has gone through rapid development with several major milestones (**Figure 1**) [3–5,9–28].

Over the past decade, CRISPR-Cas systems have emerged as a transformative genome editing technology, revolutionizing biological research and enabling unprecedented applications in both basic and applied studies. However, the traditional DSB-based CRISPR-Cas9 system is limited by off-target effects and unpredictable editing outcomes (*e.g.*, large deletions, chromosomal rearrangements, and chromosome loss) [2], which have driven the rapid development of novel CRISPR systems with enhanced accuracy, precision, and versatility. Here, we aim to provide a comprehensive overview of CRISPR technology and its applications, with a particular emphasis on the development of new techniques and their applications in emerging fields.

## Primary CRISPR-Cas systems targeting DNA and RNA

The CRISPR-Cas system typically consists of two parts: a Cas protein cleaving nucleic acids, and a guide RNA (gRNA) binding to the Cas protein. gRNA directs Cas protein to protospacer adjacent motif (PAM) for cleavage. CRISPR-Cas systems are classified into two classes and further divided into six types (types I–VI), based on the protein components of effector modules, the structure of loci, and the organization of *cas* genes [29]. Class 1 systems (types I, III, and IV) involve multiple Cas subunits that assemble into a complex to mediate interference. In contrast, Class 2 systems (types II, V, and VI) rely on a single, larger Cas protein with multiple domains [30,31]. Compared to Class 1, Class 2 is more popular due to its simplicity and efficiency.

### Cas9 in the type II systems targeting DNA

Cas9 in the type II systems is characterized by a distinct two-lobed structure comprising a recognition (REC) domain and a nuclease (NUC) domain. The REC domain contains the bridge helix motif along with REC1, REC2, and REC3 subdomains, while the NUC domain consists of two components — RuvC and HNH, to separately cleave the two strands of DNA [32]. Cas9 proteins from different bacteria have different sizes and distinct catalytic efficiencies, and recognize different PAM sequences. SpCas9 originating from *Streptococcus pyogenes* is the most widely used nuclease and recognizes the PAM of NGG (N is A, T, C, or G) sequence [33]. After the DSBs occur, the cells repair the damaged DNA through non-homologous end joining (NHEJ) pathway or homology-directed repair (HDR) pathway. The NHEJ pathway serves as the primary mechanism for DNA repair in eukaryotic cells and functions through the direct ligation of DNA break ends. This process often generates small insertions or deletions (indels), which induce frameshift mutations in the original DNA sequence, and lead to the inactivation of the target gene [34,35]. In contrast, HDR is an accurate DSB repair pathway that utilizes an exogenous homologous DNA template to direct the repair process [36].

### Cas12a in the type V systems targeting DNA

A few years after the discovery of Cas9, Cas12a (Cpf1) from Type V CRISPR-Cas systems was identified and similarly adapted for use in genome editing in 2015 [18], which catalyzes DSBs in genomic DNA using its RuvC domain. Although Cas12a also contains a NUC domain, this domain has no hydrolytic function. Unlike Cas9, Cas12a does not rely on tracrRNA for activation, but requires only crRNA [37]. When Cas12a recognizes the target sequence containing a 5′-terminal TTTV (V is G, C, or A) PAM, Cas12a utilizes its single RuvC domain catalytic site to cleave both strands within the PAM-distal region of the target site, and subsequently degrades additional single-stranded DNA (ssDNA) substrates in a *trans*-cleavage manner, which produces a staggered end different from the blunt end produced by Cas9 [38]. Compared to Cas9, Cas12a is miniature and easier to deliver, and it contains an RNase domain that can process pre-crRNA and facilitate simultaneous targeting of multiple sequences [39]. However, Cas12a remains less widely used than Cas9 for genome editing. The PAM sequence of Cas12a is enriched in T, which is not as prevalent in the genome as the NGG sequence of SpCas9, and the non-target cleavage caused by *trans*-cleavage activity of Cas12a also limits its applications *in vivo*.

### Cas13 in the type VI systems targeting RNA

Distinct from the DNA-targeting Cas9 and Cas12a systems, Cas13 from Type VI CRISPR-Cas systems demonstrates single-stranded RNA (ssRNA)-targeting activity. It features two higher eukaryotes and prokaryotes nucleotide-binding (HEPN) domains, enabling it to cleave RNA substrates directed by crRNA. Following the discovery of DNA-targeting editing systems, Feng Zhang’s team first discovered an RNA-targeting nuclease Cas13a (C2c2) in 2016, which can be used for RNA interference and detection in eukaryotic cells [20]. Subsequently, they found that Cas13b (C2c6) plays a comparable role in RNA hydrolysis [40]. After that, RfxCas13d (CasRx) originating from *Ruminococcus flavefaciens* was discovered [41], which has a smaller size than Cas13a and Cas13b, making it easier to deliver [42]. The targeting activity of Cas13d is independent of the prototype spacer flanking sequence (PFS), which is similar to the PAM sequence of Cas9. Cas13d exhibits significantly higher knockdown efficiency compared to Cas13a and Cas13b [43,44], but the collateral cleavage activity of Cas13d greatly limits its clinical application, as it was demonstrated to lead to lethal cytotoxicity in mice [45]. Nevertheless, in contrast to the Cas9 and Cas12a systems, the Cas13 system possesses several distinct advantages. It enables reversible editing, operates without PAM constraints, and is applicable to the editing of non-coding RNAs, including long non-coding RNAs (lncRNAs), which have been verified to outperform short hairpin RNA (shRNA) for gene knockdown *in vitro* [46,47]. At present, several variants of Cas13 have been engineered to regulate the non-specific collateral cleavage activity of the Cas13 system [48], enhancing its ability to specifically target and transiently reduce RNA levels.

## Technological innovations of CRISPR-Cas systems

**Figure 2:**
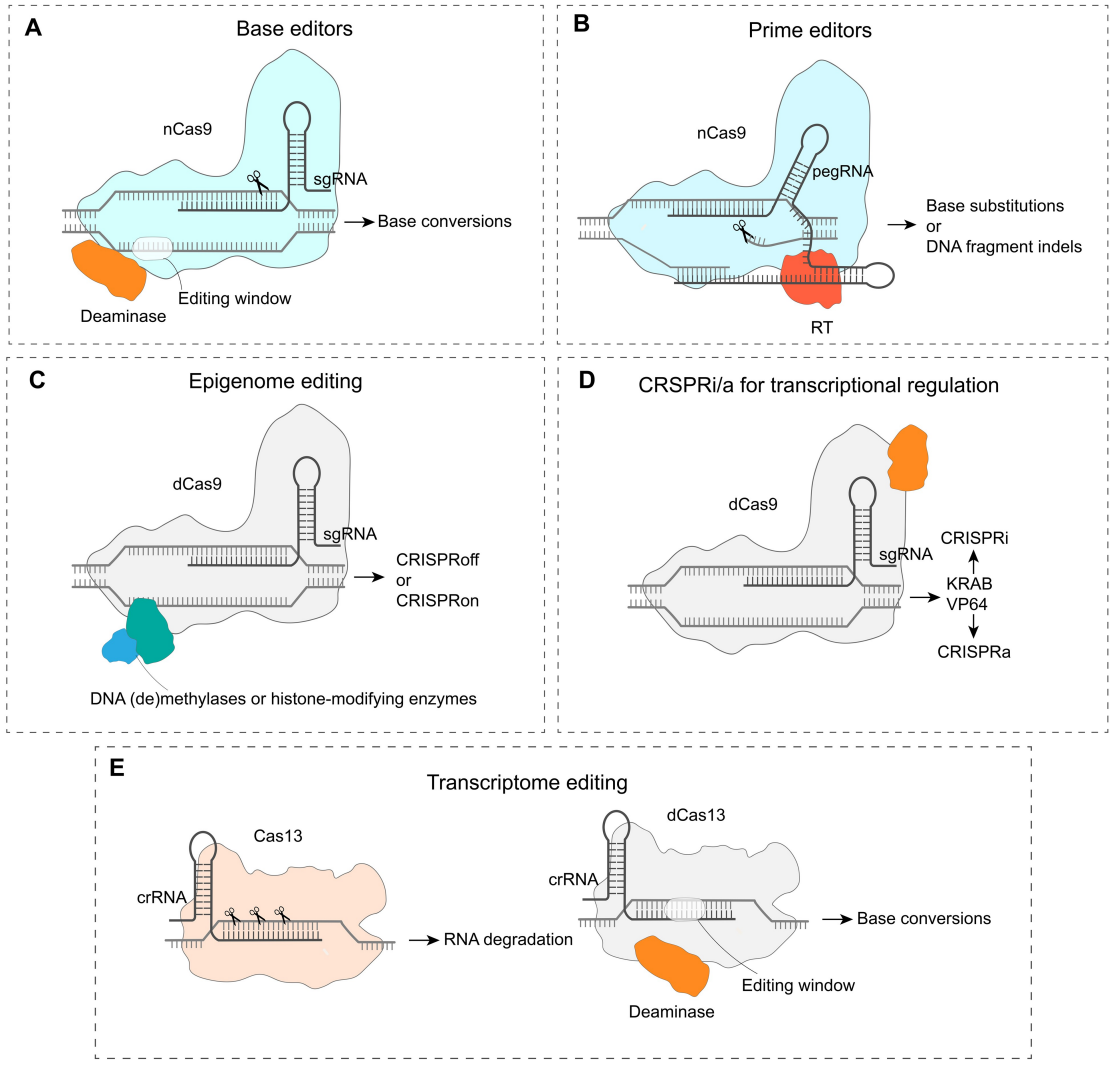
An overview of technological development of CRISPR-Cas systems **A**. Base editors: this system employs a fusion of nCas9 with nucleobase-modifying enzymes to directly convert nucleotide base. **B**. Prime editors: this system combines nCas9 with a RT and uses a pegRNA to enable the base substitutions or DNA fragment indels. **C**. Epigenome editing: this approach fuses dCas9 with DNA (de)methylases or histone-modifying enzymes to enable targeted chromatin modifications at specific genomic locations, leading to CRISPRoff (gene inactivation) or CRISPRon (gene activation). **D**. CRISPRi/a for transcriptional regulation: this system regulates gene expression by fusing dCas9 with transcriptional inhibitors (CRISPRi-KRAB) or transcriptional activators (CRISPRa-VP64). **E**. Transcriptome editing: this approach utilizes RNA-targeting Cas13 nucleases to edit RNA sequences. nCas9, nickase Cas9; pegRNA, prime editing guide RNA; indel, insertion and deletion; RT, reverse transcriptase; dCas9, deactivated Cas9.

The adaptation of CRISPR-Cas systems into efficient genome editing tools has significantly propelled both basic and applied research. However, they are constrained by several critical factors, such as editing accuracy (specificity for the target site), editing precision (producing the exact desired editing outcome), and targeting scope. To address these constraints, a range of innovative strategies has been devised to enhance the efficiency and flexibility of CRISPR-Cas systems (**Figure 2**).

### Cas9 variants

To date, the prototypical SpCas9 protein continues to be the most extensively utilized genome editing tool, attributed to its inherently high activity and specificity. However, this natural Cas9 protein has various limitations in the editing process, which significantly affects its practical application. Improved Cas9 protein variants have been developed to achieve better editing results, including reducing the off-target activity, controlling editing outcomes to improve safety, and broadening the targeting scope.

To mitigate off-target effects of Cas9, Feng Zhang’s team employed a strategy using a Cas9 nickase mutant in combination with two gRNAs that target adjacent sites to induce targeted DSBs. This approach increases the number of bases specifically recognized for target cleavage, thereby effectively reducing off-target activity [49]. Afterward, many high-fidelity variants have been engineered to significantly reduce off-target rates and retain high efficiency, such as enhanced-specificity SpCas9 (eSpCas9) [50], further eCas9 (FeCas9) [51], and SuperFi-Cas9 [52]. To improve editing precision and safety, Jiazhi Hu’s team generated a novel enzyme Cas9TX by fusing an optimized human recombinant TREX2 protein with SpCas9, to prevent chromosome translocation and other structural abnormalities during genome editing [53]. Cas9TX significantly improves editing safety and is currently considered the safest variant. In addition, the absence of a PAM sequence in the vicinity of the target site frequently poses a significant challenge to achieving precise and accurate editing. Several SpCas9 variants with expanded PAM recognition have been engineered. In 2018, David Liu’s team developed an SpCas9 variant called xCas9 that can recognize a broader range of PAM sequences, including NG, GAA, and GAT [54]. xCas9 was used in the genome editing of rice [55] and base editing of human adult stem cells [56]. Subsequently, many SpCas9 variants were developed to expand the PAM scope, such as SpCas9-NG for NG PAMs [57], SpG for NGN, and SpRY for NRN (R is A or G) or NYN (Y is C or T) [58]. In addition to SpCas9, variants derived from other species, such as ScCas9 and Nme2Cas9, further broaden PAM recognition (**Table 1**) [55,57–62]. However, expanded targeting scope often compromises specificity, resulting in the increase of off-target effects [63]. In practical operations, there is often a trade-off between these two factors.

**Table 1:** Variants of Cas9 with broader PAM recognition

Variant	PAM	Year	Ref.
xCas9	NG, GAA, and GAT	2018	[55]
SpCas9-NG	NG	2018	[57]
SpG	NGN	2020	[58]
SpRY	NRN and NYN	2020	[58]
iSpyMac	NAA	2020	[59]
ScCas9	NNG	2020	[60]
eNme2-T/C and eNme2-C.NR	N_4_ (T/C) N	2023	[61]
SpRYc	More flexible PAM recognition than SpRY	2023	[62]

*Note*: xCas9 is an SpCas9 variant. eNme2-T/C and eNme2-C.NR are Nme2Cas9 variants. N is A, C, G, or T; R is A or G; and Y is C or T. CRISPR, clustered regularly interspaced short palindromic repeats; Cas, CRISPR-associated protein; PAM, protospacer adjacent motif; SpCas9, *Streptococcus pyogenes* Cas9; ScCas9, *Streptococcus canis* Cas9; Nme2Cas9, *Neisseria meningitidis* Cas9.

### Base editors

Base editors (BEs) are constructed by fusing a catalytically inactive nickase Cas9 (nCas9) to a nucleotide deaminase enzyme, and are versatile for inducing targeted point mutations. Unlike traditional methods, they do not rely on the generation of DSBs or HDR templates, which makes precise genome editing possible in HDR-deficient cells. In 2016, David Liu’s team developed the first cytosine BE (CBE) by fusing APOBEC1, a cytosine deaminase enzyme, to nCas9 to convert C-to-T [19]. Subsequently, this team also created an adenine BE (ABE) that facilitates the conversion of A to G by fusing an adenosine deaminase to nCas9. In comparison with CBEs, ABEs exhibit greater editing efficiency and reduced off-target effects [22]. Since the initial development of CBEs and ABEs, they have undergone multiple design refinements to enhance activity and minimize off-target edits caused by deaminase. Additionally, the range of base editing has been extended to include A-to-C [64], A-to-Y [65], and C-to-G [66] conversions. In addition, to convert dual bases at the same target site, adenine and cytosine deaminases were fused to nCas9, generating a dual adenine and cytosine BE (A&C-BEmax) [67]. Further optimization resulted in enhanced versions, such as eA&C-BEmax and hyA&C-BEmax, which have achieved higher efficiency [68].

Currently, the continued development of BEs has resulted in versions with significantly enhanced on-target activity and expanded compatibility with various Cas effector proteins [69]. In parallel, as a great tool in protein 3D structure prediction, artificial intelligence (AI) technology like AlphaFold3 has been used to develop a series of new BEs [70–72]. Due to the predictable and precise editing results, BEs have been utilized for genome-wide knockout and mutant screening [73], and are well-suited for therapeutic corrections of diseases resulting from single-point mutations [74,75]. The first BE developed in the clinic is VERVE-101, targeting liver *PCSK9*-silencing, which is delivered to the liver in the form of lipid nanoparticles (LNPs). In addition to VERVE-101, there have also been several BEs in preclinical research phase [76]. Addressing the challenges in clinical therapy involves two key considerations: (1) reduce off-target effects and (2) enhance delivery efficiency to target tissues, so more efforts need to be made in the two aspects in the future.

### Prime editors

Similar to BEs, prime editors (PEs) are also based on CRISPR-Cas9 systems and enable targeted point mutations and precise small indels without relying on the DSB generation and HDR pathway. PEs are composed of two main elements: a protein component and a prime editor gRNA (pegRNA). The protein component typically consists of nCas9 fused with a reverse transcriptase (RT) domain. Meanwhile, the pegRNA features an sgRNA scaffold along with an RNA extension that encodes the desired mutation and serves as the template for the RT. In 2019, David Liu’s team fused RT with nCas9 containing an inactivated HNH domain to develop a prime editing system. This system is capable of reverse transcribing the RNA template sequences into the ends of the broken DNA strands, enabling base substitutions or DNA fragment indels [24]. Subsequently, the team developed a system employing a pair of engineered pegRNAs (epegRNAs) to enhance the precision of the editing targets [77]. The development of dual-pegRNAs greatly improves prime editing efficiency and enables larger fragment editing, enriching the application of prime editing in mammals and plants, such as homologous 3′ extension mediated prime editor (HOPE) [78], PRIME-Del [79], bi-direction prime editing (Bi-PE) [80], twin prime editing (twinPE) [81], prime editor nuclease-mediated translocation and inversion (PETI) [82], and engineered plant prime editor (ePPE) [83]. In addition, David Liu’s team has generated a series of PEs (PE6a-g) with novel RTs or Cas9 domains through phage-assisted evolution and protein engineering technology [84], achieving high editing efficiency *in vivo*. This team also edited the hematopoietic stem and progenitor cells (HSPCs) from patients with sickle-cell disease (SCD) *in vitro* using prime editing and observed successful phenotype rescue in mice after transplantation. The edited HSPCs maintained their capacity to home and engraft within the bone marrow [85].

Compared to other CRISPR systems, PEs are attractive due to the improved flexibility and specificity. However, several obstacles remain to be tackled, with the most pressing issue being low efficiency. Employing more active nCas9 or RT variants through rational design, *in vitro* evolution, and the incorporation of additional or combinatorial functional protein domains offers a promising avenue to boost the efficiency of prime editing.

### Epigenome editing

Epigenetic regulation has a significant impact on gene expression, CRISPR-based epigenome editing can induce targeted epigenetic alterations that do not alter the DNA sequence but are stably inherited across cell generations, providing a promising alternative to conventional genetic modification [86]. Catalytically inactivated Cas9 is incapable of cleaving DNA, but can still bind to DNA sequences under the guidance of sgRNA. By introducing point mutations in the RuvC1 (D10A) and HNH (H840A) domains of Cas9, the deactivated Cas9 (dCas9) protein can be produced [86]. Combining dCas9 with different epigenetic regulators, such as DNA (de)methylases or histone-modifying enzymes, can carry out epigenome editing on target sites, leading to CRISPRoff (gene inactivation) or CRISPRon (gene activation) [87]. It has been reported that in a mouse model of renal fibrosis, the expression of the dCas9-TET1 catalytic domain (dCas9-TET1cd) system results in the demethylation and activation of promoters for two anti-fibrosis genes, thereby alleviating renal fibrosis symptoms [88]. In addition, the fusion of deactivated Cas13d (dCas13d) with various epigenetic regulators can be used to study RNA epigenetic modifications. In 2021, Yang Li’s team and others developed the reengineered *N*^1^-methyladenosine (m^1^A) modification valid eraser (REMOVER) through dCas13d fused with the catalytic activity domain of ALKBH, to catalyze the m^1^A demethylation reaction on the targeted messenger RNA (mRNA). REMOVER can efficiently erase m^1^A modifications on any type of RNA, including mRNA *PRUNE* and lncRNA *MALAT* [89].

Based on these techniques, the study of specific genes becomes more flexible. CRISPRoff and CRISPRon enable stable and reversible control of transcription at targeted DNA sites, potentially allowing for the modulation of gene expression in clinical settings without the need for continuous expression of the editing components. However, since Cas9 can temporarily bind to off-target sites guided by the gRNA, the resulting epigenetic modifications might alter the transcriptional status of these off-target genes, which requires further research to fully understand their consequences.

### CRISPR interference and CRISPR activation for transcriptional regulation

The CRISPR interference (CRISPRi) system, which combines dCas9 with the transcriptional repression domains, such as KRAB or SRDX, can direct the repressive complex to the promoters of target genes, thereby disrupting transcriptional initiation or elongation [17,90]. Likewise, the CRISPR activation (CRISPRa) system, which pairs dCas9 with transcriptional activators like VP64 and its variants, can enhance gene expression levels [91,92]. Using CRISPRi and CRISPRa systems, mRNA transcription can be transiently modulated without modifying the genomic sequence. In parallel, the two systems have also brought great convenience to the research of non-coding sequences [93]. For example, lncRNAs are difficult to knock out and knock down. CRISPRi can inhibit lncRNA expression at the transcriptional level without considering its subcellular localization and secondary structure. Joanna Bereta’s team used the inducible dCas9-KRAB-MeCP2 system to achieve near-complete transcriptional suppression of the lncRNA *NORAD* in HeLa cells [94]. Meanwhile, CRISPR-dCas9 is of great significance for studying the function of enhancers. Jesse Engreitz’s team developed a new technique called CRISPRi-FlowFISH, which can be utilized to investigate the regulatory connections between enhancers and their target genes, thereby providing insights into the functions of the non-coding regions of the genome [95]. In addition, CRISPRi and CRISPRa have been widely employed for conducting loss-of-function and gain-of-function genetic screening in recent years and for identifying genes associated with various cellular functions and pathways [96]. Compared to epigenetic modulators that induce stable transcriptional changes, CRISPRa and CRISPRi can complement epigenome editing methods by enabling tunable and transient transcriptome engineering. As more efficient Cas variants and effectors are developed, along with improved modulators, the capabilities of CRISPRi and CRISPRa for genetic transcriptional modulation are expected to achieve significant advancements in the future.

### Transcriptome editing

The identification of ssRNA-targeting Cas13 enzymes has spurred the creation of molecular tools for precise transcriptome editing. Since RNA is typically temporary, transcriptome editing provides a potentially safer alternative to DNA editing. RNA degradation and gene knockdown mediated by Cas13a, Cas13b, and Cas13d demonstrate higher efficiency and specificity compared to RNA interference, which makes them promising for therapeutic applications, including the treatment of nervous system diseases and infections caused by pathogenic viruses [97–99]. In addition, a high-fidelity Cas13Y (hfCas13Y) system has been developed by Hui Yang’s team, which can degrade *MECP2* mRNA and reduce protein levels in the brains of humanized *MECP2* transgenic mice. This intervention restored dysregulated gene expression and ameliorated behavioral deficits [100]. Similar to DNA BEs, dCas13 has been combined with the deaminase ADAR2 to create RNA BEs. In 2017, Feng Zhang’s team developed the first RNA single-base editor called RNA editing for programmable A-to-I replacement (REPAIR), which uses dCas13 to change A to I at specific sites on RNA [23]. Subsequently, RNA editing for specific C-to-U exchange (RESCUE) was developed to convert C to U [101]. In addition to altering RNA bases, there are also some editors engineered to modify RNA. For example, dCas13 fused to the METTL3 methyltransferase domain is able to perform *N^6^*-methyladenosine (m^6^A) modification [102,103], which can regulate transcript abundance and alternative splicing [104]. Moreover, the REMOVER method described above enables targeted demethylation of m^1^A sites on specific RNA transcripts [89]. Directly editing RNA instead of DNA is a safer method because it avoids permanent changes or genomic off-target effects. Therefore, transcriptome editing based on CRISPR-Cas13 systems has expanded the potential for gene function research and disease treatments, both of which benefit from transient or reversible genetic modulation.

## Applications of CRISPR-Cas in basic research

Advancements in CRISPR-based genome editing technology have led to numerous applications in fundamental biological research. CRISPR-Cas systems allow us to modulate the expression of genes in a variety of animal models to study their functions and cellular mechanisms, such as cultured cells [105], organoids [106], and animal models [107–109]. In the meantime, CRISPR-Cas systems have been developed for nearly all major crops, including rice, wheat, and cotton [110], and multiplex genome editing has also been successfully adapted to the plant science field [111]. Beyond genome editing in animals and plants, CRISPR-Cas systems also enable the development of large-scale genetic screening methods and synthetic genetic recording models for lineage tracing.

### Large-scale genetic screening

CRISPR-based large-scale genetic screening (CRISPR screening) is performed using a gRNA library. This library consists of a large number of gRNAs that target different genes [112], which are typically packaged into lentiviral vectors and introduced into cells at a low multiplicity of infection (MOI), ensuring that each cell receives at most one gRNA [113]. Because the gRNA delivered by lentivirus can randomly integrate into the genome, it serves as a barcode to identify which gRNAs are enriched following screening, thereby pinpointing the differentially expressed genes [114]. At the end of the CRISPR screening, the next-generation sequencing (NGS) data reflect the abundance of gRNAs that can be used to identify gene targets with specific functions [115]. A variety of methods for CRISPR screening, such as knockout screens [116,117], CRISPRi [118,119], CRISPRa [120,121], BEs [122], PEs [123], and Cas13 [124], have enabled the identification of many genes with previously unknown functions. In addition, CRISPR screening is also applied to explore functional lncRNAs [125] and circular RNAs (circRNAs) [46].

Conventional CRISPR screening methods are designed to identify genes associated with readily selectable phenotypes, such as cell growth, drug resistance, or fluorescence-activated cell sorting (FACS). As single-cell RNA sequencing (scRNA-seq) technology has advanced, single-cell CRISPR (scCRISPR) screening methods have emerged. These methods integrate scRNA-seq with CRISPR screening, allowing for transcriptome profiling of individual cells following genetic perturbations within a complex cellular mixture [126]. Methods for scCRISPR screening, such as Perturb-seq, CRISP-seq, and Mosaic-seq, all insert a barcode into the gRNA vector to trace related gene, and obtain transcriptome information of cells with the same barcode [127–131]. In parallel, CRISPR screening combined with imaging technology and spatial transcriptomics can be utilized to pinpoint gene localization and conduct cell phenotypic analyses. For instance, Perturb-map, a spatial functional genomics screening technology that integrates spatial transcriptomics, has been employed to identify genes associated with tumor growth and immune composition [132]. Overall, high-throughput screens based on CRISPR-Cas systems provide more flexible and efficient methods than previously used techniques, such as RNA interference screens. CRISPR screening methods are expected to advance further with the ongoing discovery and engineering of new Cas nucleases, as well as the development of technologies that improve the sensitivity of these assays and their readouts.

### Lineage tracing

Depicting the history, current state, and future fate of cells is a core objective in developmental biology, with lineage tracing being the gold standard to elucidate the relationships between progenitor cells and their descendants. CRISPR barcodes, which consist of indels generated by CRISPR-Cas9 systems, can be used to specifically label cells. When combined with scRNA-seq, these barcodes enable the reconstruction of developmental lineage trees. To date, multiple techniques have been developed and applied in animal models, such as zebrafish and mice. In 2016, Jay Shendure’s team developed the first lineage tracing method using CRISPR barcodes in zebrafish called genome editing of synthetic target arrays for lineage tracing (GESTALT). This approach employs CRISPR-Cas9 to sequentially introduce and accumulate mutations in the sgRNA-targeted DNA barcode over multiple rounds of cell division, thereby labeling cells. Lineage relationships are then clarified by examining the shared mutation patterns among cells. GESTALT was used to trace the cell lineage of adult zebrafish organs [133]. To extend lineage tracing to mammals, Fernando Camargo’s team developed the CRISPR array repair lineage tracing (CARLIN) system, which induces CRISPR cleavage to generate barcodes at any developmental stage in mice. They applied this technology to investigate the clonal heterogeneity of hematopoietic stem cells (HSCs) [134]. Compared with other lineage tracing methods, CARLIN can analyze the lineage of single cells and transcriptome information simultaneously and achieve spatiotemporal-specific labeling. However, the number of barcodes that CARLIN can produce is limited. By fusing terminal deoxynucleotidyl transferase (TdT) with Cas9, doxycycline-inducible CRISPR array repair lineage tracing (DARLIN) was developed to introduce more base insertions in three distinct genomic loci, greatly improving the traceable mutation diversity [135].

CRISPR-based lineage tracing methods combine information on clonal relationships with single-cell transcriptomics. Compared to traditional lineage tracing using fluorescent labeling, these new methods have significantly enhanced resolution and accuracy, allowing for the detailed investigation of the phylogenetic basis of biological processes involving multiple cell generations at an unprecedented level of detail and scale [136]. However, CRISPR barcodes combined with scRNA-seq lack the capability of real-time imaging and lose the spatial information. Several studies have merged lineage tracing with spatial transcriptomics using imaging-based methods. For instance, iTracer was developed to examine clonality and lineage dynamics during cerebral organoid development [137]. The integration of CRISPR barcodes, spatial multi-omics, and imaging technologies in the future will significantly facilitate the study of animal developmental trajectories.

## Applications of CRISPR-Cas in clinical medicine

The emergence of CRISPR-Cas systems has transformed modern biology by offering an easy, efficient, and versatile platform for DNA editing and a highly efficient tool for clinical medicine, such as genetic diagnosis and therapeutic gene correction for disease treatment.

### Genetic diagnosis

Owing to their programmability and capacity to target specific nucleic acid sequences within genomes, CRISPR-Cas systems have emerged as promising tools in molecular diagnostics [138]. The currently established CRISPR-Cas-based genetic diagnosis is primarily classified based on three Cas proteins — Cas9, Cas12, and Cas13. Due to their notable collateral activity on nonspecific oligonucleotides and the ability to amplify the original signal to a certain extent, Cas12 and Cas13 proteins are utilized more widely in CRISPR-Cas-based genetic diagnosis (**Table 2**) [139–149].

**Table 2:** Primary diagnostic systems based on Cas12 and Cas13

Diagnostic system	Effector	Amplification	Sensitivity	Application	Year	Ref.
SHERLOCK	Cas13a	RPA	High	Detection of specific strains of Zika and Dengue viruses, pathogenic bacteria, and SNPs	2017	[139]
DETECTR	Cas12a	RPA	High	Detection of human papillomavirus	2018	[140]
CDetection	Cas12b	RAA	High	Detection of DNA viruses	2019	[141]
HOLMES	Cas12a	PCR	High	Detection of DNA and RNA viruses	2018	[142]
HOLMESv2	Cas12b	LAMP	High	Detection of SNPs, RNA viruses, human mRNAs, and circRNAs	2019	[143]
CARVER	Cas13a/Cas13b	RT-qPCR	High	Detection of RNA viruses	2019	[144]
CARMEN	Cas13	PCR/RPA	High	Scalable and multiplexed detection of pathogens	2020	[145]
iSCAN	Cas12a	RT-LAMP	High	Detection of RNA viruses	2020	[146]
iSCAN-V2	Cas12b	RT-RPA	High	Detection of RNA viruses	2022	[147]
SAHARA	Cas12a	/	Medium	Multiplexed detection of DNA and RNA	2023	[148]
EXTRA-CRISPR	Cas12a	RCA	High	Detection of miRNAs	2023	[149]

*Note*: SHERLOCK, specific high-sensitivity enzymatic reporter unlocking; DETECTR, DNA endonuclease targeted CRISPR *trans* reporter; CDetection, Cas12b-mediated DNA detection; HOLMES, hour low-cost multipurpose highly efficient system; CARVER, Cas13-assisted restriction of viral expression and readout; CARMEN, combinatorial arrayed reactions for multiplexed evaluation of nucleic acids; iSCAN, *in vitro* specific CRISPR-based assay for nucleic acids detection; SAHARA, split activators for highly accessible RNA analysis; EXTRA-CRISPR, endonucleolytically exponentiated rolling circle amplification with CRISPR-Cas12a; RPA, recombinase polymerase amplification; RAA, recombinase-aid amplification; (q)PCR, (quantitative real-time) polymerase chain reaction; LAMP, loop-mediated isothermal amplification; RT, reverse transcription; RCA, rolling circle amplification; SNP, single nucleotide polymorphism; circRNA, circular RNA; miRNA, microRNA; mRNA, messenger RNA.

When guided to their target nucleic acids by crRNA, Cas12 and Cas13 effectors activate collateral cleavage activity, nonspecifically degrading nearby ssDNA or ssRNA. In a typical CRISPR-Cas detection setup, an ssDNA or ssRNA reporter labeled with a fluorophore–quencher (FQ) pair is introduced. Upon collateral cleavage, this reporter releases a fluorescence signal, establishing a correlation between the concentration of target molecules and the intensity of the fluorescence output [150]. In 2017, Feng Zhang’s team first created the specific high-sensitivity enzymatic reporter unlocking (SHERLOCK) system based on CRISPR-Cas13a system [139], and a year later, they released a second version of SHERLOCK called SHERLOCKv2. The new detection system achieves relative quantification and a 3.5-fold improvement in sensitivity [151]. Subsequently, the first Cas12a-based diagnostic system called DNA endonuclease-targeted CRISPR *trans* reporter (DETECTR) was developed [140]. Based on these groundbreaking advances, more molecular diagnostic systems based on Cas12 and Cas13 have been generated and utilized for detecting virus infections, such as Cas12b-based DNA detection (CDetection) [141], one-hour low-cost multipurpose highly efficient system (HOLMES) [142], and Cas13-assisted restriction of viral expression and readout (CARVER) [144]. Currently, nucleic acid detection primarily relies on polymerase chain reaction (PCR) technology. In comparison, CRISPR-based detection technology exhibits a series of notable advantages, such as the ability to conduct highly sensitive and specific detection of targets under mild conditions. In the future, more Cas protein variants can be developed to minimize off-target effects or optimize PAM sequences to further broaden the detection scope.

### Gene therapy

Owing to its potential for correcting disease-causing mutations through repair mechanisms found in most cell types, CRISPR-Cas technology is a highly promising therapeutic tool for a variety of genetic disorders. In late 2023, the approval of the first CRISPR-based human therapy Casgevy in Europe, the UK, and the USA marked a new era for genome editing in disease treatment. SCD and transfusion-dependent thalassemia (TDT) are caused by various mutations in the *HBB* gene, which are the targets of Casgevy. By disrupting an erythroid enhancer in the *BCL11A* gene in CD34^+^ HSCs, fetal γ-globin (HBG) expression can be reactivated, providing a viable solution for treating SCD and TDT [152]. For treating acquired immune deficiency syndrome (AIDS) and acute myeloid leukemia (AML), CRISPR-Cas9 was employed to knock out the *CCR5* gene in donor HSPCs, which were then transplanted into patients with HIV-associated AML. The patient achieved complete remission of AML four weeks post-transplantation [153]. Moreover, clinical studies based on chimeric antigen receptor (CAR) T cell therapies have also made numerous breakthrough advances and show great promise in immunotherapy for a variety of diseases [154–157]. All the abovementioned applications are *ex vivo* therapeutic approaches. In the first successful case of systemically delivered *in vivo* CRISPR gene therapy, David Lebwohl’s team utilized liver-targeted LNPs to deliver Cas9 mRNA and sgRNA. This approach was used to treat transthyretin amyloidosis caused by mutations in the *TTR* gene, and demonstrated a significant reduction in serum TTR levels in clinical trials [158]. In addition, several *in vivo* therapies for other diseases have been developed and applied [159–161].

The delivery formats of CRISPR components depend on the specific cell types involved. In *ex vivo* applications, electroporation and liposome-mediated transfection are the most commonly used methods due to their high efficiency. For *in vivo* delivery, viral vectors and LNPs are frequently employed. The main obstacles of CRISPR applied in clinical settings is the development of safe, specific, and efficient delivery methods, particularly for targeting specific organs *in vivo*. There is also a risk that many disease-causing genes have uncertain functions, and interfering with them could lead to unexpected dysfunctions in humans. With advancements in delivery methods and characterization of disease-causing genes, the range of treatable diseases via CRISPR technology will expand considerably.

## Conclusion and perspectives

**Figure 3:**
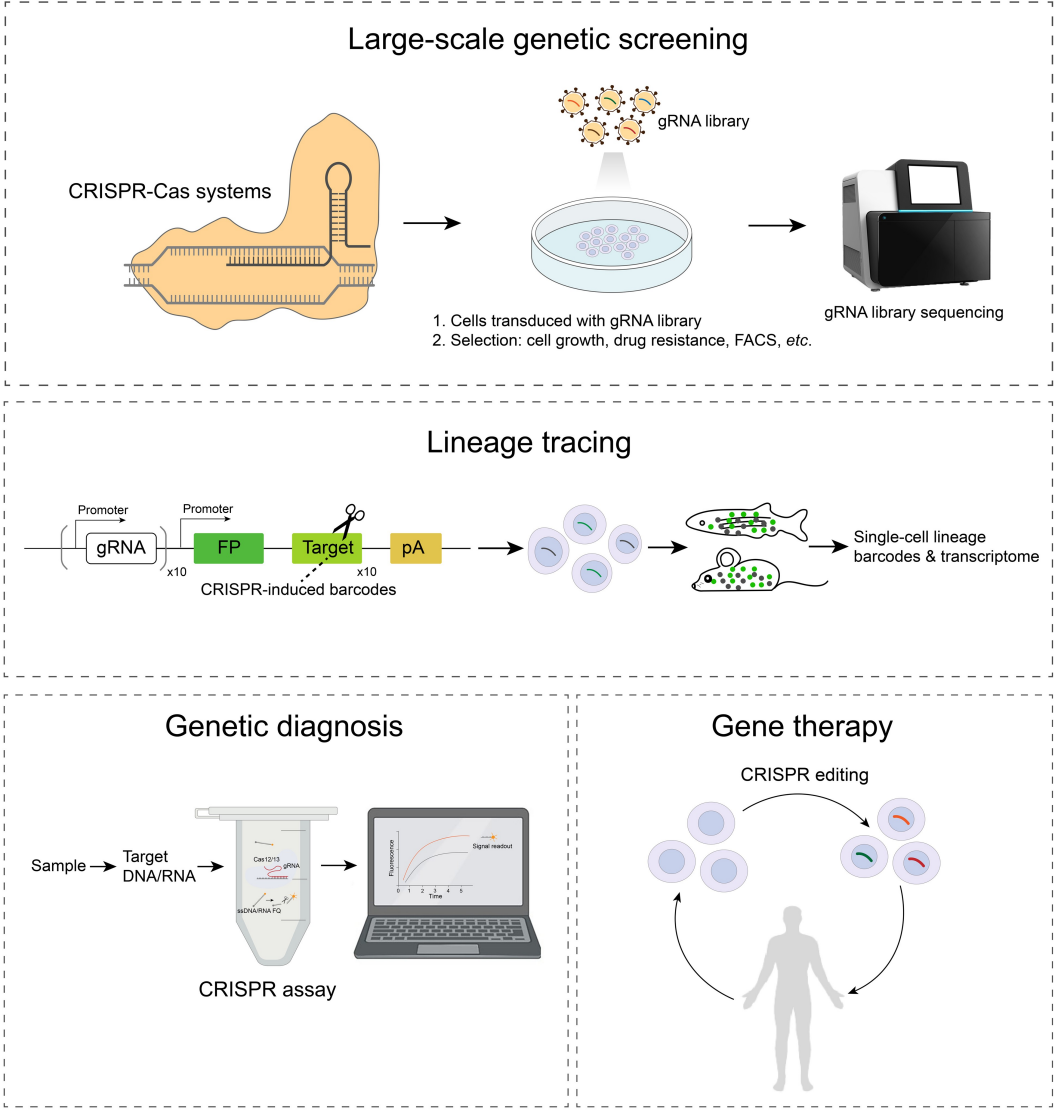
Applications of CRISPR-Cas technology CRISPR-Cas technology is widely applied in the fields of large-scale genetic screening, lineage tracing, genetic diagnosis, and gene therapy. gRNA, guide RNA; FACS, fluorescence-activated cell sorting; FP, fluorescent protein; pA, poly(A); FQ, fluorophore–quencher.

The advent of CRISPR-Cas technologies has ushered the field of genome editing into a new era. Traditional CRISPR-Cas systems, which primarily rely on site-specific DSB and endogenous repair mechanisms, have been enhanced by ongoing innovations in CRISPR technology. These advancements have transformed our capacity to manipulate genetic material. For example, BEs and PEs enable precise DNA modifications and are considered safer without DSB generation. Epigenome editing, CRISPRi/CRISPRa, and transcriptome editing offer new approaches to non-persistent editing and minimize the risk of unexpected genomic disruption. These novel systems have immensely driven the applications in basic research and clinical medicine over the past decade, including large-scale genetic screening, lineage tracing, genetic diagnosis, and disease treatment (**Figure 3**).

Currently, CRISPR technology still encounters some challenges that require further resolution, including off-target effects, targeting scope limitations, cytotoxicity, and delivery methods. Balancing high editing efficiency with minimal off-target effects continues to be an unresolved challenge. Even though various methods have been created for detecting and analyzing off-target effects, eliminating these effects altogether is still in progress. Using AI technology to predict and develop Cas variants with different structures to eliminate off-target indels is the main strategy. Moreover, several SpCas9 variants with expanded PAM recognition have been developed to address the limitations of targeting scope. The formation of DSBs in the genome can potentially cause genotoxic effects, which has spurred the development of CRISPR systems that do not depend on DSBs. Using viral vectors, such as adeno-associated virus (AAV), to deliver CRISPR-Cas is a commonly employed approach, yet it faces limitations in packaging capacity and safety [162]. The quest for safer and more efficient delivery methods remains a critical area for advancement. LNP delivery methods show significant promise for clinical applications and have been effectively used to modify disease-related targets in both humans and animal models [2]. In addition, the exploration of smaller Cas proteins, such as CasMINI and Cas12Pro, also promotes the efficiency of delivery [25,28].

Genome editing in reproductive medicine poses significant safety risks and ethical concerns. Future research must prioritize enhancing the safety and the predictability of human genome editing, while also addressing its ethical implications. While CRISPR-based gene therapy has been implemented clinically, the occurrence of cytotoxicity and mortality associated with genome editing underscores the need for greater caution regarding the unresolved issues of this technology. Accurately predicting the full range of potential side effects remains elusive. In the era of big data, leveraging existing databases to identify CRISPR-Cas proteins with enhanced editing efficiency and minimized off-target effects emerges as a highly promising approach. It is foreseeable that, with the ongoing evolution of novel editing systems, CRISPR-Cas technology is positioned to assume a more pivotal role in the prevention and treatment of human diseases.

## CRediT author statement

**Xuejing Zhang:** Writing – original draft, Writing – review & editing. **Dongyuan Ma:** Writing – review & editing. **Feng Liu:** Supervision, Funding acquisition, Writing – review & editing. All authors have read and approved the final manuscript.

## Competing interests

The authors have declared no competing interests.
